# Anesthetic Management of a Child With Rothmund-Thomson Syndrome for Major Orthopedic Surgery

**DOI:** 10.7759/cureus.95877

**Published:** 2025-11-01

**Authors:** Sara Cabete, Sara S Neves, Sónia Duarte

**Affiliations:** 1 Department of Anesthesiology, Intensive Care and Emergency, Unidade Local de Saúde de Santo António, Porto, PRT

**Keywords:** major orthopedic surgery, osteosarcoma, pediatric anesthesia, perioperative care, rare disease, recql4, rothmund-thomson syndrome

## Abstract

Children with rare diseases present unique challenges for anesthesiologists, as disease-specific factors often impact perioperative management and evidence-based guidance remains limited. Rothmund-Thomson syndrome (RTS) is a rare autosomal recessive disorder of unknown prevalence, with genetic heterogeneity and approximately 500 cases reported to date. Four genetically distinct types have been described. RTS type 2, caused by pathogenic variants in *RECQL4*, is characterized by poikiloderma, congenital bone defects, and an increased risk of cancer. Published anesthetic literature on RTS is extremely limited, with only a few case reports, none of which address major orthopedic procedures. In this context, we report the anesthetic management of a 10-year-old girl with RTS type 2 undergoing resection of a tibial osteosarcoma. This case highlights key perioperative considerations, outlines the main aspects of our anesthetic approach, and represents the first report of anesthesia for major orthopedic surgery in a patient with RTS. It emphasizes the importance for anesthesiologists to understand the multisystemic manifestations of this disorder, to engage in careful preoperative planning, as well as the need for disease-specific anesthetic guidelines to ensure safe and optimized perioperative care.

## Introduction

Children with rare diseases present unique challenges for anesthesiologists, as these conditions often have specific implications for perioperative management. In most instances, anesthesiologists cannot rely on personal experience when caring for this group of patients. Furthermore, the available information on such disorders is often limited, with sources fragmented and evidence-based medical data lacking [1,2]. While elective procedures in these patients are usually performed by specialized teams at referral centers, urgent interventions may require immediate management. Consequently, anesthesiologists must be prepared to provide safe care for these patients across all levels of medical practice. A careful pre-anesthetic evaluation, considering the disease-specific anesthetic implications, the complexity of the procedure, and the patient’s individual history, is fundamental to defining a personalized anesthesia strategy [1,2].

RTS is a rare autosomal recessive disorder of unknown prevalence (<1 per 1,000,000), with approximately 500 cases reported in the literature to date [3]. The condition exhibits genetic heterogeneity, as pathogenic variants in different genes can cause the disease [4-7]. Poikiloderma is the clinical hallmark. Other characteristic features include short stature due to pre- and postnatal growth delay, sparse scalp hair, sparse or absent eyelashes and/or eyebrows, congenital bone defects, dental anomalies, nail anomalies, hyperkeratosis, ocular anomalies, and an increased cancer risk [3,8].

Currently, four genetically distinct types are recognized, caused by biallelic pathogenic variants in *ANAPC1* (type 1), *RECQL4* (type 2), *CRIPT* (type 3), and *DNA2* (type 4). RTS type 1 is associated with poikiloderma, sparse hair, and bilateral juvenile cataracts, without increased cancer risk; growth retardation and genital, skeletal, and dental abnormalities may also be present [4]. RTS type 2 is characterized by poikiloderma, congenital bone defects, and an increased risk of osteosarcoma in childhood and skin cancer later in life [5]. RTS type 3 is associated with poikiloderma, sparse hair, short stature, skeletal defects, microcephaly, neurodevelopmental delay, and seizures [6]. RTS type 4 presents with generalized poikiloderma, severe short stature, ocular anomalies, sparse hair, craniofacial dysmorphisms, photosensitivity with bullae, dystrophic nails, and bone anomalies [7].

Diagnosis is based on clinical findings, particularly the age of onset of the skin lesions, as well as their distribution and the development of poikiloderma. A multigene panel including *RECQL4*, *ANAPC1*, *CRIPT*, *DNA2*, and other relevant genes may help to identify the genetic cause [3,8]. The recent identification of three new genes associated with RTS (*ANAPC1*, *CRIPT*, *DNA2*) has renewed interest in elucidating the molecular and pathophysiological mechanisms of this complex disorder. Notably, these genes do not appear to share obvious functional pathways, highlighting the need for further research [8]. The clinical presentation can be heterogeneous, and some individuals remain without a molecular diagnosis [3,9,10].

Patients with RTS require long-term multidisciplinary follow-up and often need anesthesia for disease-related diagnostic and therapeutic procedures, including imaging, biopsies, dental treatments, placement of a chemotherapy port, limb surgeries, cataract extraction, and tumor excision, as well as for unrelated elective and urgent or emergent procedures.

We report the perioperative management of a pediatric patient with RTS type 2 (with identified nonsense and intronic variants in *RECQL4*) [11] undergoing resection of a tibial osteosarcoma. This case highlights key perioperative concerns and our anesthetic approach, representing the first description of anesthesia for major orthopedic surgery in this context. Although based on a patient with RTS type 2, the perioperative considerations outlined are likely to be relevant to RTS more broadly.

Given the absence of formal anesthesia guidelines for RTS, sharing clinical experience through detailed case reports is crucial to support perioperative decision-making in these patients.

## Case presentation

A 10-year-and-10-month-old girl of African origin (20 kg, 123 cm, both < 3rd percentile for age and sex) with RTS type 2, precocious puberty on triptorelin therapy, and mild intellectual disability was scheduled for resection of a right tibial osteosarcoma, followed by immediate reconstruction with fibular centralization.

Nine months prior, she presented with a limp on the right lower limb, and a tibial mass was identified on a radiograph. Shortly thereafter, she sustained a pathological fracture following a fall. Osteosarcoma was diagnosed following further imaging and biopsy. A positron emission tomography scan showed no evidence of distant metastatic disease. She subsequently underwent neoadjuvant chemotherapy prior to surgery.

**Figure 1:**
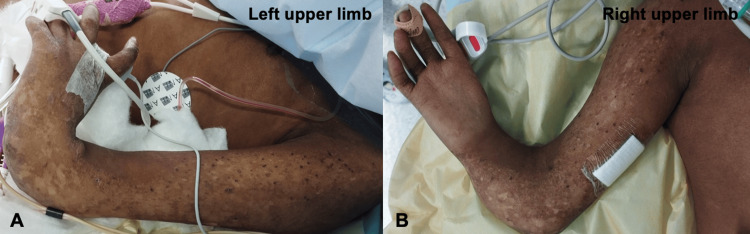
Limb anomalies in a 10-year-old girl with Rothmund–Thomson syndrome A, B: Mesomelic shortening and bowing of the upper limbs, absent thumbs, and left-hand radial deviation (prior surgical removal of hypoplastic right thumb and correction of right wrist deviation); poikiloderma

**Figure 2:**
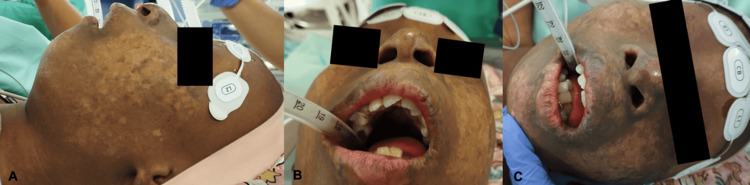
Craniofacial features in a 10-year-old girl with Rothmund–Thomson syndrome A-C: Nasal anomalies, dental anomalies, high-arched palate, micrognathia, and retrognathia; poikiloderma

She exhibited multiple syndromic features. These included short stature, low weight, and several skeletal anomalies such as bilateral forearm bone aplasia/hypoplasia, absent thumbs (the hypoplastic right thumb having been surgically removed), left radial wrist deviation (with previous surgical correction of the right side) (Figure 1), bilateral clinodactyly of the fifth fingers, dysplastic fingernails, overlapping toes, and thin calcanei. Additional features included poikiloderma involving the face, buttocks, genital region, and limbs, and craniofacial dysmorphism characterized by a long face, high forehead, depressed nasal bridge, hypoplastic nasal alae, and dental anomalies (hypoplastic, conical, and crowded teeth) (Figure 2). She had undergone multiple dental extractions due to caries and dental malformations. Airway examination revealed Mallampati class 2, good mouth opening, full neck mobility, a high-arched palate, micrognathia, and retrognathia. Sparse scalp hair, eyelashes, and thin eyebrows were previously documented and are now absent following chemotherapy. Additional figures demonstrating the syndromic features and forearm radiographs are available in a previously published case report [11].

Preoperative laboratory tests (complete blood count, biochemical profile, and coagulation studies) were within normal limits, and the echocardiogram showed no abnormalities.

The surgery was expected to last approximately four hours. The main anesthetic concerns included a potentially difficult airway, challenges in vascular access, positioning difficulties, adequate skin protection, and the risk of significant intraoperative bleeding. Perioperative blood management included blood typing, the use of tranexamic acid, and the use of a pneumatic tourniquet applied to the ipsilateral thigh during osteosarcoma resection, according to the surgical team's indication. Monitoring was planned according to American Society of Anesthesiologists (ASA) standards; monitoring also included invasive arterial pressure, processed electroencephalography (pEEG), unilateral cerebral oximetry, and the Analgesia Nociception Index (ANI). Neuromuscular monitoring was not feasible with the available equipment due to absent thumbs. The planned anesthetic technique consisted of intravenous general anesthesia combined with epidural analgesia. Inhalational induction was planned prior to peripheral intravenous access placement, with a low threshold for ultrasound-guided vascular access, including central venous catheterization if peripheral attempts failed, and ultrasound-guided arterial cannulation. Tracheal intubation with video laryngoscopy using a hyperangulated blade was also planned, with a size 2 laryngeal mask available as a rescue device. The perioperative anesthetic concerns and planned management are detailed in Table 1.

**Table 1:** Perioperative anesthetic considerations and management ANI: Analgesia Nociception Index; ASA: American Society of Anesthesiologists; IV: Intravenous; LMA: Laryngeal Mask Airway; pEEG: Processed Electroencephalography

Perioperative Concern	Clinical Considerations	Plan
Airway	Difficult airway due to craniofacial anomalies: micrognathia, retrognathia, high-arched palate, dental anomalies	Intubate with videolaryngoscope and hyperangulated blade; use LMA #2 as a rescue device
Vascular Access	Difficult venous and arterial access due to skeletal and skin anomalies: forearm skeletal anomalies, poikiloderma, fragile skin	Perform inhalational induction to minimize patient distress, anticipating multiple attempts; attempt peripheral IV first with a low threshold for ultrasound guidance, including central venous catheterization if peripheral attempts fail; perform ultrasound-guided arterial cannulation
Skeletal Anatomy	Difficulties in surgical positioning due to skeletal anomalies: left radial ray aplasia, right radial ray hypoplasia, bilateral ulnar hypoplasia with bowing, absent thumbs, left radial wrist deviation, bilateral clinodactyly of fifth fingers	Position carefully with adequate limb support; protect bony prominences
Skin	Risk of skin injury due to fragility and poikiloderma: poikiloderma involving the face, buttocks, genital region, and limbs	Use padded support surfaces; apply adhesives carefully; protect the patient's skin from all contacting devices
Blood Loss	Major orthopedic surgery with significant hemorrhagic risk (preoperative labs normal)	Preoperative blood typing; place an arterial line for invasive blood pressure and blood sampling; insert two peripheral IV cannulas; administer IV tranexamic acid before surgery; use a pneumatic tourniquet for as long as possible
Intraoperative Monitoring	Expected surgical duration of 4 hours; risk of significant blood loss	Monitor according to ASA standards; invasive arterial pressure, processed EEG, unilateral cerebral oximetry, ANI; guide rocuronium clinically (neuromuscular monitoring not feasible); insert an indwelling urinary catheter
Anesthesia and Analgesia	Need for multimodal analgesia for major orthopedic surgery	Administer IV general anesthesia combined with lumbar epidural analgesia (sufentanil + ropivacaine); provide IV fentanyl, ketamine, paracetamol, and ketorolac
Postoperative Recovery	Management of postoperative pain and risk of complications	Monitor in high-dependency unit; provide thromboprophylaxis; followed up by the Acute Pain team with the epidural catheter in place

In the operating theater, anesthesia was induced with sevoflurane 8% in a 50:50 nitrous oxide-oxygen mixture. A 22G intravenous cannula was placed in the left greater saphenous vein at the ankle level, followed by intravenous induction with fentanyl (1 µg/kg), lidocaine (1 mg/kg), propofol (3 mg/kg), and rocuronium (1.2 mg/kg). Face mask ventilation was easy. Tracheal intubation with videolaryngoscope using a hyperangulated blade was successful at the first attempt, with a 5.5 mm endotracheal tube with a stylet. Propofol target-controlled infusion (TCI) (Paedfusor model) was then initiated and titrated under pEEG guidance.

A 22G arterial catheter was placed in the left femoral artery after unsuccessful attempts in both upper limbs, and a second 22G peripheral venous catheter was secured in the right basilic vein, both under ultrasound guidance. A lumbar epidural catheter was inserted at the L4-L5 level (Tuohy 18G x 50 mm, 3 cm advanced into the epidural space) and tunneled. An indwelling urinary catheter was also placed. The patient was positioned supine with appropriate limb support and protection of the skin and pressure points. Active intraoperative warming was applied using a forced-air warming blanket beneath the patient, with adjustments made according to measured core temperature.

Before surgery, a 15 mg/kg tranexamic acid bolus and cefazolin for prophylaxis (30 mg/kg before incision, with a 15 mg/kg repeat dose after 4 hours) were administered. Propofol TCI was guided by pEEG, and the effect-site concentration was maintained between 2.0-2.4 µg/mL. Intraoperative fluid therapy consisted of a crystalloid infusion at 4 mL/kg/h, adjusted as needed, with a 10 mL/kg crystalloid bolus after tourniquet release.

Multimodal intraoperative analgesia included fentanyl (1 µg/kg bolus as needed), ketamine (0.3 mg/kg bolus), epidural sufentanil (0.2 µg/kg bolus), and epidural ropivacaine 0.2-0.375% (5 ml boluses). Neuromuscular blockade was maintained with rocuronium boluses (0.5 mg/kg) administered upon detection of spontaneous respiratory activity. For postoperative analgesia, paracetamol (15 mg/kg), ketorolac (0.5 mg/kg), and epidural ropivacaine 0.2% (5 mL) were administered. Postoperative nausea and vomiting prophylaxis included dexamethasone (0.15 mg/kg) and ondansetron (0.1 mg/kg).

Surgery lasted 3 hours and 40 minutes, with tourniquet application during the first hour. Estimated intraoperative blood loss was 50 mL. The patient remained hemodynamically stable throughout, with adequate urine output. Final arterial blood gas analysis revealed hemoglobin 10.1 g/dL and lactate 1.2 mmol/L. Neuromuscular reversal was achieved with sugammadex (2 mg/kg, administered 90 minutes after the last rocuronium bolus), and extubation was uneventful.

The patient was admitted postoperatively to a high-dependency unit for monitoring and optimized epidural analgesia. Thromboprophylaxis with enoxaparin was initiated after surgery. The Acute Pain team provided daily follow-up while the epidural catheter remained in place. Systemic analgesia consisted of scheduled paracetamol and ketorolac as needed. Continuous epidural analgesia with ropivacaine 0.1% (5-8 mL/h) and 4 mL rescue boluses of ropivacaine 0.2% ensured adequate pain control, with no pain at rest and mild pain on movement. On postoperative day (POD) 3, the continuous infusion was switched to scheduled bolus epidural analgesia every 6 hours, supplemented as needed. At this point, ketorolac was also administered regularly, and tramadol was added on an as-needed basis. On POD 5, epidural boluses were maintained only as needed, and no doses were required. The epidural catheter was removed on POD 6.

The postoperative course was uneventful. On POD 3, she was transferred to the pediatric ward, with arterial and urinary catheters already removed. She remained there for five additional days and was discharged home on POD 8 with planned outpatient orthopedic follow-up and rehabilitation.

## Discussion

RTS patients may display few or many of the associated clinical features, and the severity of these features can also vary. Clinical suspicion is usually raised due to ectodermal manifestations, as this compartment is affected in most RTS patients. The first signs involve the skin, hair, nails, and teeth. Poikiloderma is the main diagnostic hallmark. It starts with facial erythema, associated with some swelling and, less often, blisters, usually developing within the first year of life. The lesions then spread to the extremities (extensor areas first, followed by flexor areas) and buttocks, generally sparing the trunk, and eventually progress to the characteristic features of poikiloderma, consisting of persistent telangiectatic lesions, reticulated hypo- and hyperpigmentation, and punctate atrophy. Early aging of the skin and palmoplantar hyperkeratosis can also occur. Hair anomalies include sparse, brittle, thin or absent scalp hair, and sparse eyelashes and/or eyebrows. The nails are commonly dystrophic or poorly formed. Dental anomalies are diverse, and an increased incidence of caries has been observed. Ocular manifestations include bilateral cataracts, often of juvenile onset, corneal abnormalities, glaucoma, retinal atrophy, iris dysgenesis, and strabismus [3,8-10].

The skeletal system is particularly affected, with congenital bone defects ranging from overt to subtle (detectable only on radiographs). They include frontal bossing, saddle nose, radial ray defects, malformed ulnae, absent or hypoplastic thumbs, hypoplasia/agenesis of the patella, syndactyly, metaphyseal striations, and osteopenia [3,8-10].

Gastrointestinal manifestations, sometimes presenting with feeding difficulties in infancy (occasionally requiring tube feeding), include chronic emesis, anal atresia, esophageal or pyloric stenosis, and diarrhea that typically resolves in early childhood. Respiratory manifestations, such as bronchiectasis, and endocrine or hematologic abnormalities, including hypogonadism, anemia, and leukopenia, have also been reported in some patients. Low birth weight, slow weight gain, and linear growth deficiency are present in at least two-thirds of RTS patients. They are proportionately small (for both weight and height) without asymmetry [3,8-10].

RTS patients have an increased risk of cancer, with osteosarcoma prevalence around 30% (isolated or multicentric) and a median age of 11.5 years, earlier than in non-syndromic cases. Metastatic osteosarcoma is a leading cause of mortality in RTS patients. Other tumors include skin cancers, predominantly squamous cell carcinoma (around 5% prevalence), as well as myelodysplasia, acute myeloid leukemia, malignant fibrous histiocytoma, basal cell carcinoma, and Bowen's disease. RTS should be considered in all patients with osteosarcoma, particularly when associated with skin changes [3,8].

Neurocognitive development is generally unaffected, except in patients with biallelic *CRIPT* variants [3,8].

As with other rare diseases [1,2], whatever the indication for anesthesia in patients with RTS, assessment of the pathophysiology of the disease (its manifestations and documentation of the current multidisciplinary follow-up), determination of whether the risk of anesthetic complications is increased (e.g. difficult airway), and identification of which specific precautions need to be foreseen (such as additional preoperative investigations or post-operative admission to intensive care or a high dependency unit) should be undertaken. In these patients, it also remains critical not to overlook the fundamentals of a standard pre-anesthetic evaluation: family and patient history, allergies, recent upper airway infection, etc. Previous medical and surgical histories should also be considered, as they are commonly extensive [1,2].

Information from the patient’s physicians, like the pediatrician, though not directly related to anesthesia, can inform the anesthetic plan [1,2]. Other potential sources of information are published reports accessed through medical literature databases and textbooks, but anesthesia-specific information can be scarce. There are also online free-access resources, like Orphanet [3], National Organization for Rare Disorders (NORD) [12], and Online Mendelian Inheritance in Man (OMIM) [4-7]. They offer information on the genetic, clinical, pathological, and therapeutic aspects of rare diseases, but anesthesia is rarely considered [1,2]. The most useful Internet-based tool available on anesthetic management of rare diseases is Orphananesthesia [13], although to date, it does not have an entry on RTS (as of August 2025).

Available information in the literature and online should be interpreted with caution, as its scientific value is of variable quality and weak when based solely on a few case reports. Ultimately, it remains up to the anesthesiologist to make a clinically relevant synthesis of all the information available and to define an anesthetic strategy [1].

In our case, a primary anesthetic concern was the patient’s upper limb malformations and skin manifestations, which required careful positioning with appropriate padding and meticulous protection of the fragile skin. In this setting, all electrodes, wristbands, straps, and cuffs should be applied carefully, preferably using non-adhesive or padded devices. Limb alterations also made vascular access difficult, and as the patient had no pre-existing venous access, inhalational induction with a face mask was chosen to avoid distress, since multiple attempts were anticipated. Ultrasound guidance was planned to assist with vascular access (venous and arterial).

Airway management was another critical consideration. In addition to characteristic dental anomalies, our patient had a high-arched palate, micrognathia, and retrognathia. In these patients, airway management can also be challenging due to typical facial features such as frontal bossing and saddle nose. Appropriate airway equipment and experienced practitioners are therefore mandatory, as in our case. Careful airway manipulation is also important to avoid dental injury, particularly when dental anomalies are present.

Anesthesiologists should also consider other perioperative risks associated with RTS. Respiratory symptoms should be assessed. Patients with gastrointestinal involvement may be prone to dehydration, electrolyte disturbances, and malnutrition. The impact of preoperative fasting needs to be considered, and careful, timely evaluation and optimization should be carried out. Preoperative serum electrolytes should be monitored, and adequate hydration ensured. Preoperative investigations should also include a full blood count, given the potential for hematological abnormalities.

To our knowledge, only two other case reports on the anesthetic management of RTS have been published, describing anesthesia for an esophagogastroduodenoscopy and for cataract surgery [14,15]. The main issues highlighted in these reports included the risk of difficult airway, challenges with vascular access, the need for skin protection, and the importance of thorough preoperative evaluation. Our experience was similar, although for a longer and more invasive procedure, highlighting the same key considerations. It also emphasized careful limb positioning due to the patient’s significant upper limb anomalies, the impossibility of neuromuscular monitoring, and aspects of anesthetic management specific to the surgical procedure in question. Table 2 provides a concise comparison of anesthetic management in our case and the two previously reported cases [14,15], based on the available data.

**Table 2:** Comparison of anesthetic management in three patients with Rothmund-Thomson syndrome ETT: Endotracheal Tube; HDU: High-Dependency Unit; IV: Intravenous; LMA: Laryngeal Mask Airway; PACU: Post-Anesthesia Care Unit; PONV: Postoperative Nausea and Vomiting; POD: Postoperative Day; pEEG: Processed Electroencephalography; RTS: Rothmund-Thomson Syndrome; TCI: Target-Controlled Infusion; TIVA: Total Intravenous Anesthesia

	Tibial osteosarcoma resection and reconstruction with fibular centralization	Esophagogastroduodenoscopy	Cataract surgery
Patient	10-year-old child, RTS type 2	11-year-old child, RTS type not specified	7-year-old child, RTS type not specified
Anesthetic Technique	IV anesthesia combined with epidural analgesia	Total IV anesthesia	Balanced general anesthesia
Preoperative Concerns	Difficult airway (micrognathia, retrognathia, high-arched palate, dental anomalies), vascular access, positioning challenges, skin fragility, bleeding risk	Gingival hypertrophy with prolonged bleeding (routine laboratory tests within normal limits; hematology consult obtained), fragile skin	Difficult airway (Mallampati IV, limited mouth opening), increased oral bleeding and aspiration risk due to hypodontia and gingival hypertrophy, risk of dehydration, electrolyte imbalance, and hypoglycemia
Vascular Access	Inhalational induction prior to IV placement; two peripheral IV cannulas (22G): first attempt successful at the left greater saphenous vein (ankle), second cannula in the right basilic vein under ultrasound guidance; arterial line (22G) under ultrasound guidance, placed in the left femoral artery after multiple failed attempts in the upper limbs	24G peripheral IV secured with non-adhesive dressing	Peripheral IV access was unsuccessful in the ward after multiple attempts; successfully placed after inhalational induction
Airway	Tracheal intubation with videolaryngoscope and hyperangulated blade after neuromuscular relaxation with rocuronium; LMA available as a rescue device	Intubation following neuromuscular relaxation with atracurium	LMA used; backup devices included various ETT sizes, LMAs, stylets, bougies, and videolaryngoscope
Positioning / Skin Protection	Supine with limb support; protection of pressure points and fragile skin	Not detailed	Soft cotton padding under blood pressure cuff; careful protection of pressure points
Intraoperative Management	Propofol TCI; multimodal analgesia with epidural ropivacaine and sufentanil, IV fentanyl, ketamine, paracetamol, and ketorolac; dexamethasone and ondansetron for PONV prophylaxis; tranexamic acid; fluid therapy at maintenance rate with a bolus after tourniquet release; multimodal monitoring including invasive arterial pressure and pEEG; rocuronium was guided clinically, as neuromuscular monitoring was not possible due to absent thumbs	Midazolam and fentanyl for sedation; diagnostic phase under TIVA with propofol and ketamine, with spontaneous ventilation maintained; intubation for esophageal dilatation; fluid therapy with Ringer’s lactate; ondansetron for PONV prophylaxis; extubated uneventfully after a 30-minute procedure	Propofol and fentanyl; sevoflurane for maintenance; spontaneous ventilation maintained; fluid therapy with 3.3% dextrose + 0.3% NaCl; 35-minute procedure
Postoperative	HDU admission; thromboprophylaxis; follow-up by Acute Pain team: epidural analgesia with continuous infusion then scheduled boluses, with rescue doses as needed; paracetamol, ketorolac, and tramadol for systemic analgesia; transfer to ward POD 3; epidural catheter removal POD 6; discharge POD 8	Not detailed	PACU admission; stable recovery; transferred to ward after reaching adequate sedation score; uneventful postoperative course and discharge the next day

## Conclusions

This case adds to the limited anesthetic literature on RTS, providing the first description of anesthesia for major orthopedic surgery in a patient with RTS type 2. It illustrates the perioperative complexity of this syndrome, including potential airway difficulties, challenging vascular access, skin fragility requiring meticulous protection, and skeletal anomalies affecting patient positioning. These features reflect the multisystemic nature of RTS and emphasize the importance of thorough preoperative assessment. This report highlights the importance of an individualized anesthetic plan addressing both RTS-related and procedure-specific challenges, including perioperative blood management, tailored monitoring, and the choice of anesthetic technique, which contributed to a safe perioperative course and positive outcome.

These insights support the need for structured, disease-specific guidelines, such as those promoted by Orphananesthesia, to guide anesthesiologists in similar scenarios, and to promote standardized, safe perioperative care for patients with RTS.
